# Acceptance of Open Preperitoneal Repair in Inguinal Hernia Surgery Delphi-Consensus After an Anonymous International Survey Among European Hernia Society Members

**DOI:** 10.3389/jaws.2024.13840

**Published:** 2025-02-04

**Authors:** Ralph Lorenz, Willem Akkersdijk, Gabriel Paiva De Oliveira, Tim Warren, Marc Soler

**Affiliations:** ^1^ Hernia Center 3+CHIRURGEN, Berlin, Germany; ^2^ Department of General and Abdominal Surgery, Clinic for General and Abdominal Surgery, Medical University Brandenburg an der Havel, Neuruppin, Germany; ^3^ Clinic for General and Abdominal Surgery, St Jansdal Hospital, Harderwijk, Netherlands; ^4^ Abdominal Wall Team, Department of Surgery, Hospital Garcia de Orta, Almada, Portugal; ^5^ Abdominal Wall and Upper GI Team, Hospital Lusíadas Lisboa, Lisboa, Portugal; ^6^ Triducive Partners Limited, St Albans, United Kingdom; ^7^ Service de Chirurgie Viscérale et Digestive, Clinique Saint-Jean, Cagnes-sur-Mer, France

**Keywords:** inguinal hernia, groin hernia, open preperitoneal techniques, Delphi-consensus, tailoring

## Abstract

**Introduction:**

For years, the Lichtenstein technique was the gold standard for open repair, but several open pre-peritoneal techniques have developed since the fifties of the 20th century that offer some benefits over the Lichtenstein technique in terms of post-surgical incidence of pain. Since the 2023 update of the International HerniaSurge Guidelines, open preperitoneal mesh techniques have been an acceptable alternative, providing available expertise and competence with at least equal results as Lichtenstein repair.

**Aim:**

The aim of this project is to understand the views of surgeons regarding the approach to inguinal hernia repair and determine best practice principles for optimal surgical outcomes.

**Methods:**

Using a modified Delphi method, a panel of experts developed 43 Likert scale statements across six key domains. These statements were used to develop an online survey distributed to surgeons in Europe involved in inguinal hernia repair. The threshold for consensus was set *a priori* at 75%.

**Results:**

A total of 202 responses were received from surgeons involved in inguinal hernia repair over three rounds of survey. After the initial survey round, seven statements were revised and reissued for a further round. At the conclusion of the survey phase, 31 of the 38 remaining statements achieved consensus (of which 13 achieved ≥90% agreement). From these results, the panellists developed a set of 3 recommendations to help define principles for optimal approach to inguinal hernia repair. Accordingly Open preperitoneal techniques seems to be an alternative to Lichtenstein technique if expertise is available and should be included in a tailored concept. Knowledge of anatomy, Education and Training in open preperitoneal techniques is crucial for the acceptance of these techniques.

**Conclusion:**

The proposed set of recommendations provides some principles for surgeons to consider when selecting an approach to inguinal hernia repair, ensuring good patient outcomes in a practical and cost-effective manner.

## Introduction

Groin hernia repair is one of the most common surgeries performed globally on around 20 million people annually [1, 2]. There are two main types of groin hernias: femoral hernias and inguinal hernias [3]. The majority of inguinal hernias are symptomatic, and surgery is the only curative treatment. Even amongst the minority of patients who are asymptomatic and managed with a watch-and-wait approach, surgery will be required within 5 years in approximately 70% of cases.

Hernia repair may be undertaken as open (“classical”) or laparoscopic [4] surgery. In addition, the specific surgical technique chosen for a repair is influenced by several factors: the need to use a synthetic mesh for reinforcement of the repaired posterior wall, individual patient factors (such as obesity), primary or recurrent hernia, patient preference, and surgeon experience.

Both open and laparoscopic techniques are associated with low rates of recurrence, but laparoscopic surgery is generally associated with lower rates of chronic pain (when compared to some open techniques). Chronic pain after inguinal hernia surgery can occur in up to 10%–12% of cases [5].

While surgery is successful in most cases, recurrence of hernia affects over 10% of cases, with 57% occurring within 10 years of surgery [6].

Open preperitoneal mesh techniques are a long-standing and globally accepted option for the treatment of inguinal hernias. Since the fifties of the 20th century, numerous surgical techniques have been developed. Since the 2023 Update of the Herniasurge Guidelines, an open preperitoneal mesh technique has been an acceptable alternative, providing available expertise and competence with at least equal results as Lichtenstein repair [7]. Regarding the use of mesh, international HerniaSurge guidelines recommend the use of a mesh in the majority of cases, noting that “Although there is strong evidence that mesh repair is superior to non-mesh, there are cases in which a non-mesh repair can be suggested” [7].

Both open and laparoscopic techniques are associated with low rates of recurrence, but laparoscopic surgery is generally associated with lower rates of chronic pain. However, laparoscopic surgery requires access to endoscopic equipment with suitably trained surgeons and is therefore associated with greater costs and a steeper learning curve [8, 9].

Open tension-free mesh repair (Lichtenstein) is a popular technique due to its easy reproducibility by non-specialist surgeons [9]. Whilst this technique is associated with low recurrence and complication rates, there are concerns over reported chronic post-surgical pain [10]. Over time, alternative open repair techniques (e.g., open new simplified totally extraperitoneal, “ONSTEP”; TransREctus sheath Preperitoneal, “TREPP”; TransInguinal Preperitoneal repair, “TIPP” and Minimal Open PrePeritoneal repair, “MOPP”) have strived to offer the simplicity of the Lichtenstein method while reducing the risk of chronic post-surgical pain. Open approaches have been used safely and effectively for a number of years, but their evidence is limited, and the choice of approach may be based on surgeon experience [5]. Due to difficulties in conducting an RCT in surgery, there is often a lack of comparative data to determine the optimal approach. An alternative, albeit with weaker evidence, is to capture the expert opinion of European surgeons regarding aspects of inguinal hernia repair [11].

The aim of this project is to understand the views of surgeons regarding approach to inguinal hernia repair, and determine best practice principles for optimal surgical outcomes.

## Methods

Following an independent facilitator (Triducive Partners Ltd.)’s review of available literature, a steering group of European surgeons experienced in inguinal hernia repair (see Author list) convened in December 2022 to discuss surgical methods employed in inguinal hernia repair. The steering group was selected based on published research and experience in inguinal hernia surgery.

This project was funded by BD Medical Ltd. by supporting the costs of the methodological process, which was performed by Triducive Partners Ltd. Ian Walker and Tim Warren are employees of Triducive Partners Ltd. and acted as facilitators during the expert group discussions to identify key topics and to generate the consensus statements. The survey distribution was supported by the European Hernia Society (EHS), and Triducive Partners Ltd. performed an independent analysis of the results.^1^

**FIGURE 1:**
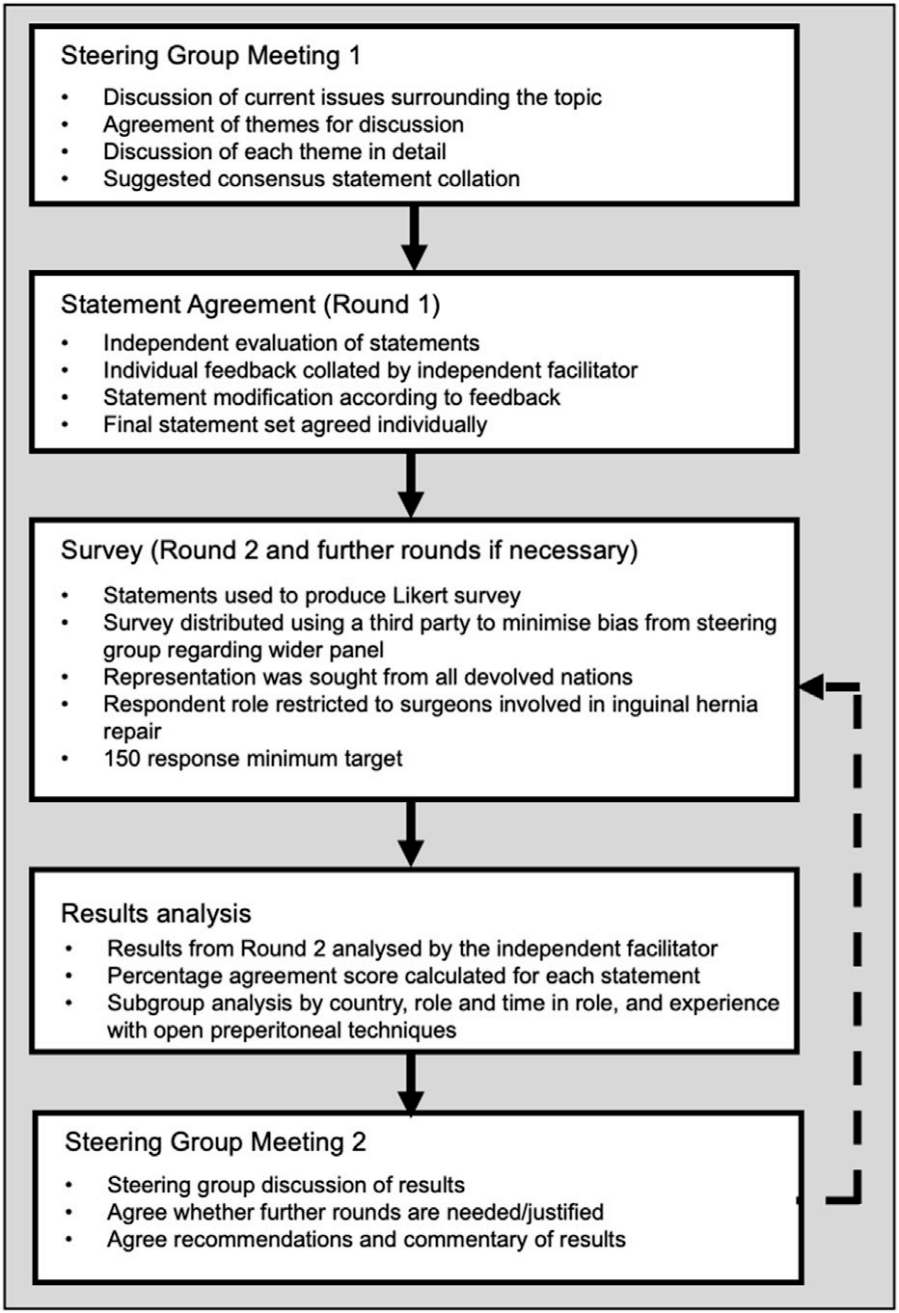
Modified Delphi study design.

Using a modified Delphi methodology (see Figure 1) guided by the independent facilitator, the steering group identified six main domains of focus:

1. Indication and diagnosis
2. Selection of patients (the right patient for the right procedure)
3. Technical considerations and best practice
4. Management of complications and risk
5. Wider impact of various surgical approaches
6. Education support required to support outcomes

These domains were each discussed by the steering group and 42 statements were initially agreed. The steering group members reviewed the statements independently to remove, add or change any statements. Suggestions were upheld if either they provided more clarity to a statement or were agreed by a simple majority of the group. The resulting 41 statements were then used to develop the final agreed statement set for wider testing. This constituted the first round of the process.

The survey was distributed using a snowball method by the steering group members to inguinal hernia surgeons and professional societies, including the European Hernia Society (EHS), for distribution to their membership. A further review by EHS for accuracy and balance resulted in a finalised set of 43 statements. A consequence of using this approach is that it is not known how many individuals the survey was sent to, and so a response rate cannot be calculated. In October and November 2023, this anonymised survey with these statements was sent out to EHS members. Each online questionnaire response entered an MS-Excel-based program. The results were evaluated as part of a Delphi Consensus (>90% agreement = strong consensus, >75–90% consensus, <75% no consensus).

The survey presented each statement along with a 4-point Likert scale (“strongly disagree,” “tend to disagree,” “tend to agree,” and “strongly agree”) to allow respondents to indicate their corresponding level of agreement. The survey also captured some demographic data for further analysis. Demographic data captured included surgical speciality, country, time in a professional role, and general experience with open pre-peritoneal inguinal hernia repair (and specifically familiarity with open new, simplified, totally extraperitoneal (ONSTEP) technique). All responses collected from respondents based in Europe were included in the final analysis.

Stopping criteria were established as the minimum target of 150 responses in Round 2, with 90% of statements passing the threshold for consensus and a threshold for consensus set at 75% (a widely accepted threshold [12]). Consensus was then further defined as “strong” at ≥75% and “very strong” at ≥90%. If these criteria were not met, the steering group had the opportunity to modify those statements that fell below the threshold for consensus and reissue them for subsequent rounds of the survey.

A statement of consent was included at the start of the survey and consent was implied by completion and submission of the survey. As this study only collected the anonymous opinions of surgeons and no patient specific data was captured, ethical approval was not sought.

Completed surveys were collated and analysed by the independent facilitator to produce an arithmetic agreement score for each statement. This information was then reviewed by the members of the steering group to determine what recommendations and conclusions could be developed based on the responses received.

## Results

During the first round of consensus testing with the members of the steering group, the initial set of 42 statements was critically reviewed to determine the final set of statements for broader testing. From this first round, one statement was removed, 18 statements were modified and agreed upon, no new statements were included, and 23 statements were agreed upon for inclusion without modification, producing a final set of 41 statements for testing with a broader panel of experts. Distribution by EHS was pursuant to an additional review for balance and accuracy. This review resulted in the addition of two statements and further modification of 10 existing statements, which the steering group agreed upon, resulting in a final set of 43 statements.

221 (180 + 41) surgeons responded after the first, second and third rounds of the survey.

Surgeons from 37 countries worldwide took part in this survey.

The second round of testing comprised the broader body of European inguinal hernia surgeons.

Of this second round, 180 responses were collected, of which 18 were from non-European respondents, which were not included in the final results set. Of the 162 responses included in the analysis, 105 were from general surgeons, 26 gastrointestinal surgeons, 15 abdominal wall surgeons, 14 hernia surgeons and 2 colorectal surgeons. Responses were received from 27 European countries, the largest responder groups being Portugal (n = 44), Germany (n = 27), Italy (n = 19), France (n = 17), and Spain (n = 14); all other country responses were ≤5.

Results from Round 2 showed very strong agreement (≥90%) in 13/43 (30%) statements, strong agreement (<90% and ≥75%) in 16/43 (37%) statements and failure to achieve consensus agreement threshold in 14/43 (33%) statements. Of those that failed to achieve consensus, five statements were removed, seven were reworded, and it was agreed that the remaining two would be reported but not revised. Seven statements were retested with the more comprehensive panel in Round 3.

**FIGURE 2:**
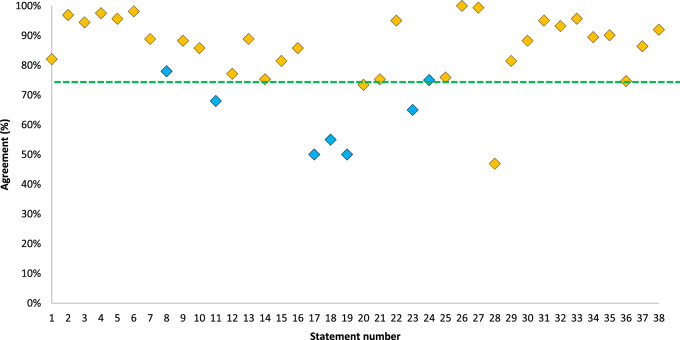
Combined consensus scores across 39 statements. The green line represents the threshold for consensus (75%). Points in orange = Round 2 (n = 162) accepted results, Blue = Round 3 (n = 40) results.

The final results from Rounds 2 and 3 show consensus agreement for 31 of the 38 remaining statements (of which 13 achieved ≥90% agreement), consensus agreement was not achieved for 7/38 statements (n = 202), results are shown in Table 1 and Figure 2.

**TABLE 1:** Defined consensus statements and corresponding levels of agreement attained (R2: n = 162, R3: n = 40) Statements with Strong consensus , consensus , No consesus .

No.	Statement	Agreement
**Topic A: Indication and diagnosis**		
1	The indication for surgery will be overestimated less frequently if the patient is informed and an active part of the decision-making process	82%
2	Decisions regarding surgery should always involve understanding the patient history and should take into account the patient’s views	97%
3	All other reasons for groin pain should be identified and excluded prior to any hernia surgery	94%
4	The patient must know the objectives of the surgery, and know that in some cases there will be persistence of all or part of the preoperative pain	98%
5	A physical examination is the basis for identifying and assessing inguinal hernia in patients with groin pain	96%
6	Symptomatic hernias should be operated on if the patient understands the potential outcomes and makes an informed decision	98%
7	Asymptomatic hernias should be operated on if they are at high risk of complication or there are patient factors such as significant anxiety, lifestyle requirements, etc.	89%
**Topic B: Selection of patients for open pre-peritoneal approach**		
8	Open pre-peritoneal procedures are valid options for inguino-scrotal hernias and incarcerated hernias	78%
9	Obesity is a complicating factor for inguinal hernia surgery	88%
10	Laparoscopic techniques are preferred for morbidly obese patients with inguinal hernias	86%
11	Open inguinal hernia repair offers more intraoperative opportunity for tailoring the approach in inguinal hernia repair	68%
**Topic C: Technical considerations and best practice in open pre-peritoneal approach**		
12	It is possible to change technique without conversion (that is, changing the technique without changing the plane to place the mesh) during surgery when using open pre-peritoneal surg…	77%
13	Open pre-peritoneal approaches allow for a range of anaesthetic approaches (e.g., general, local and spinal) to be used	89%
14	Open pre-peritoneal approaches reduce the need for curarisation and endotracheal intubation	75%
15	A pre-peritoneal approach covers the whole myopectineal orifice (MPO) with a prosthesis whose size is adapted to the patient and the type of hernia	81%
16	There are several open preperitoneal approaches (e.g., TREPP, TIPP, Open new simplified totally extra peritoneal (Onstep), and MOPP) leading to a pre-peritoneal mesh position that cover…	86%
17	Open preperitoneal techniques such as TREPP (TransREctus Sheath PrePeritoneal) and TIPP (TransInguinal PrePeritoneal Technique) are associated with a shorter operating time compared to Lichtenstein repair (assuming all techniques are delivered by properly trained surgeons)	50%
18	Open preperitoneal techniques are associated with an earlier return to normal daily activities compared to Lichtenstein repair (assuming techniques are delivered by properly trained surgeons)	55%
19	Open preperitoneal techniques are associated with fewer postsurgical complications compared to Lichtenstein repair (assuming techniques are delivered by properly trained surgeons)	50%
20	Open new simplified totally extraperitoneal (ONSTEP) surgery is well suited to non-obese patients with small to medium sized hernia	73%
**Topic D: Management of complications and risk**		
21	Contrary to Lichtenstein repair (where the femoral control is not always well realised), open pre-peritoneal approaches diminish the risk of missing a femoral hernia	75%
22	Contraindications relating to both the patient and the type of hernia should inform the choice of surgery provided	95%
23	Open pre-peritoneal techniques have a comparable incidence of acute post-operative pain compared to endoscopic techniques (assuming both techniques are delivered by properly trained surgeons)	65%
24	Open pre-peritoneal procedures have a comparable incidence of chronic (>1 year) post-operative pain compared to endoscopic techniques (assuming both techniques are delivered by properly trained surgeons)	75%
25	Open pre-peritoneal approaches reduce the impact of mesh nerve contact	76%
**Topic E: Education support required for open pre-peritoneal surgery**		
26	Knowledge of the surgical anatomy of the anterior and posterior approach is crucial in future education	100%
27	Poor understanding of the surgical anatomy anterior and posterior leads to poor outcomes in Inguinal Hernia Surgery	99%
28	Open pre-peritoneal surgery is easier to learn than laparoscopic surgery	47%
29	Step-by-step modules exist to help education of the techniques available for Inguinal Hernia Surgery	81%
30	Education about Inguinal Hernia Surgery should be led by experts in the field, as part of a specialized education (specific diploma) or with personalised tutoring	88%
**Topic F: Technical aspects of use**		
31	Many patients are unaware of the different available inguinal hernia surgery options	95%
32	Any hernia surgery option should involve joint decision-making discussions between the patient and surgeon	93%
33	The needs/expectations of the patient should be sought and considered before agreeing the appropriate hernia surgery	96%
34	Improving patient education about hernia surgery options would be beneficial	90%
35	The management of environmental resources should be an increasingly important component when considering hernia surgery options	90%
36	Open surgery using a local anaesthetic has a lower environmental impact than laparoscopic surgery requiring general anaesthesia	75%
37	The management of financial resources is an increasingly important component when considering hernia surgery options	86%
38	The resources of the surgical institution may impact the range of options available for use	92%

## Discussion

### Topic A: Indication and Diagnosis

There was strong agreement for all statements in Topic A, suggesting a recognition of two key principles:

1. Patients should be informed of the objectives of surgery and involved in the decision-making process and the likelihood of continuance of preoperative pain.
2. The need for and objectives of inguinal repair surgery should be based on a thorough physical examination of the patient, including careful consideration of the patient’s history and exclusion of all other reasons for groin pain.

Given that more than 2 in 3 individuals with asymptomatic hernias will require surgery within 5 years, surgery for asymptomatic hernias should be considered if there is a high risk of complication or the patient has significant anxiety or lifestyle requirements.

### Topic B. Selection of Patients for Open Pre-Peritoneal Approach

Respondents agree that open preperitoneal procedures are valid for inguinal hernia repair (S8, 78%). The respondent group also supports the use of laparoscopic techniques for inguinal hernia repair in obese patients, reflecting evidence that open surgery in these patients is associated with a greater incidence of deep surgical site infection, wound dehiscence, or return to the operating room [13].

### Topic C: Technical Considerations and Best Practice in Open Pre-Peritoneal Approach

The responder panel reached a consensus agreement regarding some of the valuable features of open pre-peritoneal repair techniques:

- They offer the possibility of changing the technique used without changing the plane used to place the mesh (S12, 77%).
- They can be used with a choice of anaesthetic approaches (as opposed to laparoscopic methods, which require general anaesthesia), potentially reducing the need for curarisation and endotracheal intubation (S14, 75%).
- Employs a mesh to cover the whole MPO, thereby reducing the risk of hernia recurrence in this area comprising Hasselbach’s triangle, deep inguinal ring, and the femoral canal (S15, 81%) [14].
- In addition, open pre-peritoneal techniques allow a complete exposure of the MPO to aid in the identification of all possible hernias in the inguinofemoral region [5].

Agreement levels for statements 17–19 are interesting; there is evidence to suggest that some open preperitoneal techniques offer advantages over Lichtenstein repairs, and conversely, there is evidence that there is no significant difference [15–17]. A meta-analysis comparing postoperative outcomes in inguinal hernia repair with transinguinal preperitoneal (TIPP) versus Lichtenstein techniques found that TIPP was associated with a lower operating time, less chronic pain, and lower rates of paresthesia compared to Lichtenstein [15].

A prospective study by Berri et al. found that ONSTEP surgery required significantly less time (42 vs. 62 min; *p* < 0.001), with fewer postsurgical complications (5 vs. 19; *p* = 0.001), and that patients resumed daily activities sooner (5.94 ± 3.9 days vs. 8.56 ± 5.14 days; *p* = 0.009) and expressed better satisfaction with the cosmetic result (*p* = 0.041) compared with Lichtenstein [16].

Analysis of statements 17–19 by responder who declared employment of open preperitoneal techniques in clinical practice shows a predictable response: those who use these techniques as their primary surgical approach (n = 13) tend to agree with these statements, those who used them “rarely” or “never” tended to disagree. This supports the idea that the optimal technique to use in inguinal hernia repair is one that the surgeon is experienced and skilled in performing.

Responses to statement 20 (which just fell short of consensus at 73%) also follow a similar pattern to statements 17–19; the specific wording of the statement may have caused some lack of agreement to clarify whether the statement was intended to provide some definition of the patient characteristics that ONSTEP is most suitable for, not establish a preference. As stated, inguinal hernia in obese patients may be better suited to laparoscopic repair; therefore, logically, open techniques are better suited to non-obese patients. The authors, therefore, suggest that ONSTEP is most suitable in non-obese patients with small-to-medium-sized hernias.

### Topic D: Management of Complications and Risk

Respondents agree that open pre-peritoneal approaches are associated with a lower risk of missing a femoral hernia, this is due to the nature of the approach which provides a good view of the ileopectineal orifice and therefore a greater opportunity to examine for femoral hernia [18].

Regarding pain, respondents did not agree overall that the incidence of acute post-operative pain was comparable between open pre-peritoneal and laparoscopic techniques (S23; 65%) but did agree that chronic pain incidence was comparable (S24, 75%). These results are representative of the limited available evidence. Whilst comparative data exists to support similar incidences of post-surgical pain for open pre-peritoneal and laparoscopic techniques [19, 20], these studies do not specifically discuss acute pain. However, they are limited to reported pain at intervals of weeks or months after surgery. In contrast, there is some evidence to suggest that laparoscopic trans-abdominal pre-peritoneal (TAPP) repair is associated with lower acute post-surgical pain than open pre-peritoneal or Lichtenstein methods [21].

There was also consensus that open preperitoneal approaches reduce the impact of nerve contact from the mesh. Anatomically, this is due to the lack of nervous structures in the preperitoneal space, which renders interaction between the mesh and nerves absent [22].

### Topic E: Education Support Required for Open Pre-Peritoneal Surgery

Throughout the results dataset, it is observed that those who use open pre-peritoneal approaches tend to answer more positively regarding statements concerning the efficacy and practical use of these techniques than those who prefer a laparoscopic approach. This is perhaps expected, and different methods should be considered for different surgical circumstances. A cohort study of 107,073 patients in the US [23] found no significant difference in complications between laparoscopic surgery and open repair under local anaesthesia, but operative time for laparoscopic repair was significantly longer (10.42 min). In summary, Meier et al. suggest that laparoscopic and open repair with local anaesthesia were reasonable options for patients with initial unilateral inguinal hernias, and the decision should be made considering both patient and surgeon factors.

Level of training, learning curve of procedure and surgeon volume are all factors that impact the outcome of a surgery [24]. Respondents very strongly agree that it is important that surgeons (particularly general surgeons) have a good knowledge of the of the surgical anatomy of both the anterior and posterior approaches and that future education should include this (S27, 100%; S28, 99%).

The lack of agreement with S28 is interesting, given that it is well established that laparoscopic techniques are associated with a steeper learning curve than open methods. HerniaSurge (2018) reports that trainees achieve proficiency after an average of 64 open repairs compared with more than 100 for laparoscopic repairs [1]. This should make open approaches a valuable choice for general surgeons or those with limited access to laparoscopic surgical resources.

### Topic F: Technical Aspects of Use

It is concerning that respondents strongly agree that many patients are unaware of different surgical options for inguinal hernia repair (S31, 95%), particularly given the very strong agreement that shared decision-making is crucial between surgeon and patient (S32, 93%), and this is supported by HerniaSurge guidelines [7]. It is recommended that local processes are in place to ensure appropriate patient education and consultation are provided and decisions are made in alignment with the patient’s individual needs.

Healthcare is responsible for almost 5% of global greenhouse gas emissions, and the growing climate crisis has been described as the greatest threat to global health in this century [25]. Respondents appear to recognise their personal responsibility for good carbon stewardship in the operating room and support the need to consider the environmental impacts of hernia surgery (S35, 90%). Anesthesia is a recognised carbon hot spot in surgery, and the use of local/regional anaesthesia is associated with lower environmental impact than general anaesthesia (particularly inhaled anaesthesia) [26].

Another concern amongst healthcare providers is the delivery of cost-effective and value-based healthcare, and surgeons agree that this is increasingly important when considering options for inguinal hernia repair (S37, 86%). Tied to this is the variation in resourcing of different surgical institutions, some of which may struggle to justify the associated additional cost/resource requirements of laparoscopic surgery, which is reported to be 41% greater in a US analysis [27]. The authors suggest that where access to laparoscopy is limited, open pre-peritoneal approaches should be considered.

### Limitations

There are limitations to the statements due to the total number of hernia specialists participating in this survey. Furthermore, surgical colleagues interested in open preperitoneal techniques could be more represented in this survey which could increase adhesions and could influence the recommendations.

## Recommendations

Based on the results obtained during the survey phase of this study and the following discussion held by the steering group, the authors offer the following set of recommendations:

1. A Tailored Approach to groin hernia surgery should include endoscopic and open techniques with and without mesh. The decision as to which technique is optimal for the patient should always be made individually, depending on the hernia and the patient’s characteristics.
2. With open preperitoneal techniques, there are alternatives to the Lichtenstein technique, if expertise is available.
3. Knowledge of anatomy, Education and Training in open preperitoneal techniques is crucial for the acceptance of these techniques.

## Conclusion

The acceptance of open preperitoneal procedures depends primarily on surgical expertise.

The advantages of open preperitoneal techniques lie primarily in the concept of intraoperative tailoring and in the selection of anaesthesia procedures up to and including implementation under local anaesthesia. In expert hands, there could also be advantages in terms of operating time, return to everyday activities and the occurrence of chronic pain.

Training plays a decisive role in this.

## Data Availability

The raw data supporting the conclusions of this article will be made available by the authors, without undue reservation.

## Footnotes

1 Triducive privacy policy can be found at https://triducive.com/privacy-policy/
